# Chromosome (re)positioning in spermatozoa of fathers and sons – carriers of reciprocal chromosome translocation (RCT)

**DOI:** 10.1186/s12920-018-0470-7

**Published:** 2019-02-01

**Authors:** Marta Olszewska, Ewa Wiland, Nataliya Huleyuk, Monika Fraczek, Alina T. Midro, Danuta Zastavna, Maciej Kurpisz

**Affiliations:** 1 Institute of Human Genetics, Polish Academy of Sciences, Strzeszynska 32, 60-479 Poznan, Poland; 2 National Academy of Medical Sciences of Ukraine, Institute of Hereditary Pathology, Lysenko Str. 31a, Lviv, 79000 Ukraine; 3 Department of Clinical Genetics, Medical University of Bialystok, Waszyngtona 13, PO Box 22, 15-089 Bialystok, Poland; 4 Department of Biotechnology and Bioinformatics, Faculty of Chemistry, Rzeszow University of Technology, Al. Powstancow Warszawy 6, 35-959 Rzeszow, Poland

**Keywords:** Chromosome topology, Familial translocation, Nuclear architecture, Reciprocal chromosome translocation, Sperm chromosomes, Male infertility

## Abstract

**Background:**

Non-random chromosome positioning has been observed in the nuclei of several different tissue types, including human spermatozoa. The nuclear arrangement of chromosomes can be altered in men with decreased semen parameters or increased DNA fragmentation and in males with chromosomal numerical or structural aberrations. An aim of this study was to determine whether and how the positioning of nine chromosome centromeres was (re)arranged in the spermatozoa of fathers and sons – carriers of the same reciprocal chromosome translocation (RCT).

**Methods:**

Fluorescence in situ hybridization (FISH) was applied to analyse the positioning of sperm chromosomes in a group of 13 carriers of 11 RCTs, including two familial RCT cases: t(4;5) and t(7;10), followed by analysis of eight control individuals. Additionally, sperm chromatin integrity was evaluated using TUNEL and Aniline Blue techniques.

**Results:**

In the analysed familial RCT cases, repositioning of the chromosomes occurred in a similar way when compared to the data generated in healthy controls, even if some differences between father and son were further observed. These differences might have arisen from various statuses of sperm chromatin disintegration.

**Conclusions:**

Nuclear topology appears as another aspect of epigenetic genomic regulation that may influence DNA functioning. We have re-documented that chromosomal positioning is defined in control males and that a particular RCT is reflected in the individual pattern of chromosomal topology. The present study examining the collected RCT group, including two familial cases, additionally showed that chromosomal factors (karyotype and hyperhaploidy) have superior effects, strongly influencing the chromosomal topology, when confronted with sperm chromatin integrity components (DNA fragmentation or chromatin deprotamination).

**Electronic supplementary material:**

The online version of this article (10.1186/s12920-018-0470-7) contains supplementary material, which is available to authorized users.

## Background

In recent years, examination of the intranuclear organization of the genome in different cell types has been well developed. In the cell nucleus, every chromosome occupies a well-defined non-random position, the so-called chromosome territory (CT). The size and location of the CT depends on the size of the chromosome, gene density, transcriptional activity, phases of the cell cycle and cell type [1, 2] Along with interchromatin compartments (ICs), topologically associated domains (TADs) constitute a fundamental structural unit and interact with elements of the nuclear matrix, creating the intranuclear architecture [1, 3–5]. Such a nuclear organization has been suggested as one of the elements adjusting epigenetic mechanisms that may regulate genome functioning. An interesting empirical model for chromosome positioning is the human sperm nucleus because of its unique chromatin compaction system. In human spermatozoa, the packaging of sperm chromatin is 4–6-fold stronger when compared to somatic cell types, resulting from the exchange of histones for protamines [6–8]. The centromeres of sperm chromosomes are directed towards the nuclear centre, where they aggregate in chromocenter/s, while telomeres tend to associate as dimers and tetramers at the nuclear periphery [6, 9–12]. Such a looped chromosome conformation seems to be critically important for normal fertilization and zygote development [13–15]. It has been suggested that paternal telomeres are the first elements of the paternal genome to interact directly with the ooplasm after fertilization. Non-random chromosome positioning in human sperm is being evaluated during meiotic stages of spermatogenesis [16–18]. Considering different criteria for sperm nuclear schematic division, the positions of all chromosomes have been presented. Additionally, similarly to diploid cells, there is also a possible association between the chromosome position and its size and gene density in spermatozoa [17, 19, 20]. Interestingly, the nuclear order of chromosomes can be altered in males with decreased semen parameters, increased DNA fragmentation and chromosomal aberrations, both structural (reciprocal translocation) as well as numerical (sperm hyperhaploidy, marker chromosome) [21–28].

Reciprocal chromosomal translocations (RCTs) are one of the most common structural aberrations in the human genome (1/700 in new-borns) and are either heritable from one of the parents or induced de novo [29]. It is estimated that 1% of infertile males exhibit RCT [30]. RCTs are present in approximately 16% of all autosomal aberrations in lymphocytes of oligozoospermic men and in approximately 4% of azoospermic cases [30]. Balanced RCTs usually do not affect the phenotype of the carrier or his sperm parameters. However, RCT carriers are at risk for an abnormal pregnancy and/or offspring with developmental disabilities, as determined by the production of genetically unbalanced gametes originating from chromosomal segregation during the pachytene stage of meiosis. In approximately half of RCT carriers, an interchromosomal effect (ICE) has been documented. This increased frequency of hyperhaploid gametes arises from disrupted disjunction of chromosomes that are not involved in a translocation and is one of the causes of reproductive failure [31–38] In familial RCT cases (i.e., father and son), men with the same RCT have concurrent meiotic segregation patterns, but, intriguingly, they mostly demonstrate different (in)fertility histories [39–47]. The reasons for such phenomena are still unknown.

The purpose of this study was to determine whether and how centromeres of chromosomes 4, 7, 8, 9, 10, 11, 18, X and Y are repositioned in the nuclei of spermatozoa of carriers of the same RCT. The observations were focused on two familial RCT cases, including fathers and sons, and the results were compared to those obtained from two groups of males: normal controls and other RCT carriers. Additionally, in a group of all collected RCTs, the role of the abnormal karyotype vs. chromatin integrity towards sperm nuclear organization order was investigated. We also aimed to assess whether the sperm nuclear topology might be diversified in relation to the frequency of genetically unbalanced gametes.

## Methods

The material for analyses consisted of spermatozoa from 13 male carriers of 11 balanced reciprocal chromosome translocations: T1–46,XY,t(1;11)(p36.22;q12.2); T2–46,XY,t(2;8)(q21;q22); T3–46,XY,t(2;10)(q13;q24.3); T4–46,XY,t(3;9)(q27;q22.3); T5–46,XY,t(4;10)(q35;q23.2); T6–46,XY,t(4;18)(q33;q22.3); T7–46,XY,t(6;14)(q21;q13.3); T8–46,XY,t(7;18)(q11.23;q12.2); T9–46,XY,t(11;13)(p15.5;q22); T10 and T11–46,XY,t(4;5)(p15.1;p12) (father and son); and T12 and T13–46,XY,t(7;10)(p21.2;q26.13) (father and son). All patients were recruited during the examination of infertility in reproductive clinics in Poznan (Poland), Bialystok (Poland), and Lviv (Ukraine). The characteristics of each RCT case, including seminology, meiotic segregation pattern, chromatin integrity status, and aneuploidy level in sperm cells, have been published elsewhere [45, 47–49] and are presented in Table 1. Ideograms of the analysed RCT cases are shown in Additional file 1: Figure S1. The control sperm cells originated from our laboratory group of healthy, fertile, male donors aged between 25 and 30 years with normozoospermia [50], in the following numbers: *n* = 7 for aneuploidy evaluation (previously published by Olszewska et al., [51]), *n* = 15 for chromatin integrity tests (previously published by Olszewska et al., [51]) and *n* = 8 for topology analysis (not published elsewhere). Ejaculated semen samples from all men were collected after 3–5 days of sexual abstinence. After liquefaction and washing in F-10 medium, sperm samples were fixed with a fresh fixative solution (methanol:acetic acid, 3:1 *v*/v, − 20 °C) and then stored at − 20 °C until further use. All men were notified about the purpose of the study, and written informed consent was obtained according to the guidelines of the Local Bioethical Committee in Poznan University of Medical Sciences (approval no. 772/15).

**Table 1:** Characteristics of reciprocal chromosome translocations (RCTs) carriers, including two familial cases (T10-T13) of fathers and sons

RCT	Karyotype	Seminal parameters^b^	% of genetically unbalanced spermatozoa^b^	% of deprotaminated spermatozoa (AB)^b^	% of spermatozoa with fragmented DNA (TUNEL)^b^	Hyperhaploidy ratio in spermatozoa (%; enumerated only results higher than Mean Control values)^b^
T1	46,XY,t(1;11)(p36.22;q12.2)^a^	N	59.6	nd	8.80	4–0.34; 8–0.40; 10–0.31; XY – 0.50
T2	46,XY,t(2;8)(q21;q22)	T	54.3	28.5	18.8	10–0.22; 11–0.14; XY – 0.73
T3	46,XY,t(2;10)(q13;q24.3)	N	50.7	66.3	35.8	7–0.22; 8–0.48; 18–0.53; XY – 0.62
T4	46,XY,t(3;9)(q27;q22.3)	AT	43.0	22.0	7.6	4–0.51; 7–0.26; 8–0.60; 11–0.18; 18–0.56; XY – 0.33
T5	46,XY,t(4;10)(q35;q23.2)	N	39.4	36.5	5.9	7–0.29; 18–0.64; XY – 0.16
T6	46,XY,t(4;18)(q33;q22.3)	N	42.3	nd	34.1	8–0.20; 9–0.32; 10–0.15
T7	46,XY,t(6;14)(q21;q13.3)	N	45.1	25.4	19.2	4–0.25; 7–0.29; 8–0.31; 9–0.37; 11–0.60; 18–0.59; XY – 0.29
T8	46,XY,t(7;18)(q11.23;q12.2)	N	65.7	45.6	5.6	11–0.22
T9	46,XY,t(11;13)(p15.5;q22)	O	45.0	22.2	19.7	10–0.21; 18–0.32; XY – 0.36
Familial case no. 1:						
T10 father	46,XY,t(4;5)(p15.1;p12)^c^	N	65.2	4.6	nd	10–0.17; 18–0.22; XX – 0.25; YY – 0.18; XY – 0.33
T11 son		T	65.6	13.7	nd	18–0.29; XY – 0.26
Familial case no. 2:						
T12 father	46,XY,t(7;10)(p21.2;q26.13)	N	37.6	nd	25.9	4–0.58; 8–0.28; XY – 0.31
T13 son		N	44.4	25.1	5.6	9–0.22; XY – 0.48
Mean RCT value ± SD		–	50.61 ± 16.78	28.99 ± 18.55*	17.00 ± 11.85*	
Mean Control value ± SD^d^		–	–	16.53 ± 7.89	8.41 ± 3.39	4–0.13; 7–0.13; 8–0.13; 9–0.12; 10–0.09; 11–0.08; 18–0.09; XX – 0.11; YY – 0.10; XY – 0.08

*N* normozoospermia, *T* teratozoospermia, *AT* asthenoteratozoospermia, *O* oligozoospermia (according to WHO, 2010)

*AB* aniline blue assay, *TUNEL* Terminal deoxynucleotidyl transferase dUTP Nick End Labeling, *nd* not done

*value statistically significant according to Mean Control value (*p* < 0.05)

^a^previously published in Midro et al., 2014, except TUNEL and hyperhaploidy results

^b^previously published in Olszewska et al., 2013, 2017 (T2-T9, T12-T13)

^c^percentage of genetically unbalanced spermatozoa previously published in Wiland et al., 2007

^d^previously published in Olszewska et al., 2014 (hyperhaploidies), 2017 (AB, TUNEL)

### Fluorescence in situ hybridization (FISH)

#### Slide preparation

Fixed sperm samples were spread onto slides, washed in 1× PBS (pH 7.0; Biomed, Lublin) and incubated in a decondensation solution (10 mM dithiothreitol (DTT; Merck), 100 mM TRIS-HCl; pH 8.5, 43 °C) for 5 to 10 min. The slides were briefly rinsed in 2× SSC (pH 7.0), air-dried and then stored at − 20 °C until the FISH procedure. The FISH procedure was prepared according to the manufacturer’s protocol (Cytocell, Cambridge, UK) with modifications as described previously [23, 47]. Briefly, for the sperm FISH experiments, only slides containing non-overlapping spermatozoa with a preserved nuclear shape and tail were selected. Additionally, the criterion of a max. 1.5-fold increased nuclei size after DTT treatment was taken into consideration. For the positioning of centromeres, two- or three-colour FISH experiments were prepared. The FISH mixture contained 2.0 μl of each α-satellite probe (5× concentrated, green and red or green, red and yellow = green+red; listed below) and was filled with a hybridization solution to a final volume of 10.0 μl (Cytocell, UK). The probes used in the experiments were centromere-specific for nine chromosomes: 4 – *locus* D4Z1 (4p11.1-q11.1), 7 – *locus* D7Z1 (7p11.1-q11.1), 8 – *locus* D8Z2 (8p11.1-q11.1), 9 – *locus* D9Z3 (9q12), 10 – *locus* D10Z1 (10p11.1-q11.1), 11 – *locus* D11Z1 (11p11.1-q11.1), 18 – *locus* D18Z1 (18p11.1-q11.1), X – *locus* DXZ1 (Xp11.1-q11.1) and Y – *locus* DYZ3 (Yp11.1-q11.1).

#### Microscopy and data analysis

Images of the FISH results were acquired with a fluorescence microscope (Zeiss D1 AxioImager, Germany) equipped with an oil-immersion 100× objective, a bandpass filter set (FITC/Texas Red/DAPI and Triple) and a CCD camera (MetaSystems, Germany). Topology analysis was performed using ISIS software fitted in measurement tools (MetaSystems, Germany). The efficiency of FISH was estimated at 98%. For each chromosome and each male, positioning in a minimum 150 of spermatozoa was analysed. The total number of spermatozoa evaluated in the study amounted to approximately 28,000 cells.

#### Localization of the centromeres

**Fig. 1:**
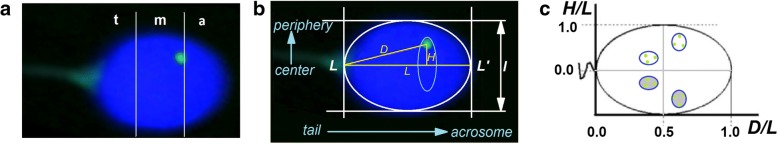
A graphical model of the linear and radial measurement techniques of centromere localization within the sperm cell nucleus. **a** Linear positioning: frequency of FISH signals counted in 3 equal parts of the nucleus, delineated along the long ‘L’ axis: ‘t’ – near the tail, ‘m’ – middle, and ‘a’ – near the acrosome. **b** Radial positioning: centromere localization – central (deep inside the nucleus) or peripheral (close to the sperm nuclear membrane), according to normalized values depicted in a coordinate system: D/L ± SE for OX axis and H/L ± SE for OY axis, where L and l – lengths of the long and short axes of the sperm nucleus; D and H – distances from the FISH signal (green point) to the tail attachment point and to the long axis; ellipse – potential mirror image of centromere localization. **c** Schematically marked chromocenter areas (groups of centromeres) with their mirror image (grey area). Radial positioning was originally determined by Zalenskaya and Zalensky [52]

The topology of chromosomes 4, 7, 8, 9, 10, 11, 18, X and Y was estimated in linear and radial measurement patterns, explained in Fig. 1, as developed by Zalenskaya and Zalensky [52] and successfully used in our previous topology studies [22–25]. In the case of chromosomes involved in RCT, in addition to centromere-specific FISH probes, probes specific for subtelomeric regions were also used (colour combinations described previously [45, 47, 48]). This colour combination allowed us to discriminate the segregants containing a set of 23 chromosomes, including normal and derivative chromosomes (spermatozoa after alternate and adjacent I segregation) (meiotic segregation patterns have been described in detail previously [45, 47, 48], and frequencies of genetically unbalanced gametes are summarized in Table 1).

#### Linear positioning

The linear positioning denotes the frequency of spermatozoa with a FISH signal in three equal regions of the sperm nucleus determined along its long axis: ‘t’ – near the tail, ‘m’ – middle part, and ‘a’ – near the acrosome (Fig. 1a).

#### Radial positioning

The radial position indicates that the centromere can be situated deep inside the nucleus (central localization) or close to the internal sperm nuclear membrane (peripheral localization). We used the graphic model described by Zalenskaya and Zalensky [52]), which includes geometric attributes of the sperm cell determining the ‘flipping coin’ mirror positions of spermatozoa on microscopic slide. The measured parameters of the sperm nucleus included the following: length of the long axis (L; from a tail attachment point to an acrosome) and short axis (l; in the widest part of the sperm nuclei), distance from the FISH signal to the tail attachment point (D) and to the long axis L (H), and the ellipsoidal shape (L/l) indicating the decondensation ratio of the nucleus (Fig. 1b). The D/L value was used to determine the position of the centromere towards the ‘tail-acrosome’ direction, with a maximum value of 1.0. The H/L value defined the position of the centromere in terms of the nuclear depth (‘centre-periphery’ criterion), with a maximum value of 0.3. The obtained values are depicted in a coordinate system as the mean D/L ± SE for the OX axis and H/L ± SE for the OY axis (Fig. 1c). The intranuclear localization areas of the analysed centromeres are shown as limited regions of the sperm nucleus – so-called chromocenters (Fig. 1c).

#### Chromosome characteristics

The characteristics of the individual chromosomes involved in each RCT are presented in Additional file 2: Table S1. All length and position values were counted in Mpz according to data from NCBI Genome Data Viewer, GRCh38.p11 (https://www.ncbi.nlm.nih.gov/genome/gdv/). A translocated segment (TS) included chromosomal fragment from the breakpoint to the end of an arm that was translocated, while an interstitial segment (IS) consisted of chromosomal region from the centromere to the breakpoint (TS + IS = chromosomal arm). The ratios of both fragments were estimated according to the length of the whole chromosomal arm involved in proper translocation.

### Statistical analysis

For statistical analysis of the linear results, a two-tailed χ^2^ test was performed with a significance level of α = 0.05. For statistical analysis of the radial results, one-way analysis of variance (ANOVA) was carried out at a significance level of α = 0.01. Estimation of the common aggregation of centromeres was performed using Ward cluster analysis and visualized as hierarchical trees with linkage to Euclidean distances. All the tests were performed using OriginLab (v. 8.5) or GraphPad Prism (v.5) software.

## Results

### Linear positioning

**Fig. 2:**
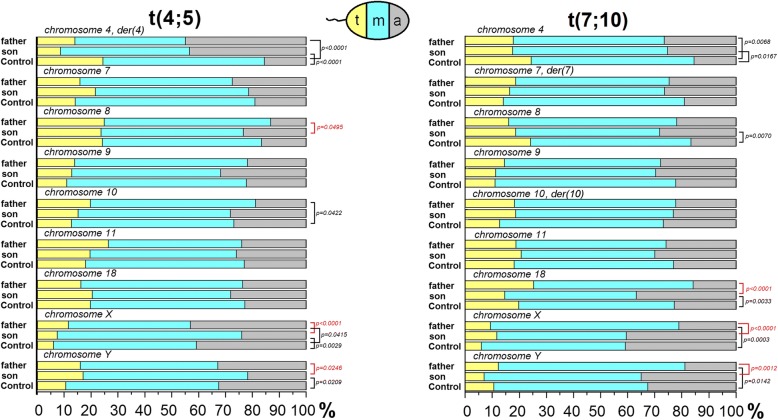
Linear positioning within the sperm nucleus of familial RCT cases vs. controls: left panel – 46,XY,t(4;5)(p15.1;p12), father T10 and son T11; right panel – 46,XY,t(7;10)(p21.1;q26.13), father T12 and son T13. The frequencies of centromeres are colour-coded according to the sperm nuclear region. Statistically significant differences (*p* < 0.05) are also depicted: between fathers and sons in red colour; mean control value (black colour). All values are presented in Table 2

The linear positioning results are presented in Table 2 and Fig. 2.

**Table 2:** Linear positioning of centromeres of chromosomes: 4, 7, 8, 9, 10, 11, 18, X and Y in spermatozoa of 13 RCT carriers

Chromosome	a - near the acrosome; m - middle; t - near the tail	T1	T2	T3	T4	T5	T6	T7	T8	T9	T10	T11	T12	T13	RCT mean value ± SD [*n* = 13]	Control mean value ± SD [*n* = 8]
		t(1;11)	t(2;8)	t(2;10)	t(3;9)	t(4;10)	t(4;18)	t(6;14)	t(7;18)	t(11;13)	t(4;5) father	t(4;5) son	t(7;10) father	t(7;10) son		
4	a	22.94	18.27	17.87	19.57	17.31	20.95	35.16	29.03	19.77	44.86	43.33	26.48	25.20	25.12 ± 5.56	15.46 ± 2.32
	m	50.15	62.50	53.99	60.14	63.46	58.785	48.35	41.94	54.65	41.12	48.00	55.73	57.22	52.38 ± 6.43	60.11 ± 3.83
	t	26.91	19.23	28.14	20.29	19.23	20.27	16.48	29.03	25.58	14.02	8.67	17.79	17.59	22.50 ± 4.61	24.42 ± 3.88
	p [T vs. C]	0.0632	0.0425^*^	0.4572	0.4084	0.4697	0.2613	< 0.0001^*^	< 0.0001^*^	0.4163	< 0.0001^*^	< 0.0001^*^	0.0068^*^	0.0167^*^		
	p [f vs. s]										0.1141		0.9483			
7	a	36.24	25.2	17.93	33.77	32.33	23.57	34.04	20.51	24.82	27.46	21.29	24.63	26.32	27.22 ± 5.99	19.01 ± 2.94
	m	49.35	56.91	69.66	48.34	47.37	58.57	53.19	64.96	60.28	56.56	57.03	56.72	57.24	56.60 ± 6.90	66.85 ± 1.67
	t	14.41	17.89	12.41	17.88	20.30	17.86	12.77	14.53	14.89	15.98	21.68	18.66	16.45	16.19 ± 2.55	14.14 ± 2.36
	p [T vs. C]	< 0.0001^*^	0.1060	0.8224	0.0002^*^	0.0001^*^	0.2125	0.0006^*^	0.9128	0.2921	0.0614	0.0568	0.0982	0.1018		
	p [f vs. s]										0.1930		0.8146			
8	a	16.05	32.22	13.51	20.95	21.43	14.29	28.57	14.14	26.32	13.13	23.29	21.90	28.13	21.59 ± 6.57	16.57 ± 2.48
	m	57.31	54.44	55.41	50.00	55.19	63.27	51.19	53.54	48.03	61.95	52.97	61.98	53.13	54.86 ± 4.65	59.21 ± 1.76
	t	26.65	13.33	31.08	29.05	23.38	22.45	20.24	32.32	25.66	24.92	23.74	16.12	18.75	23.55 ± 6.11	24.22 ± 3.11
	p [Tvs. C]	0.8517	< 0.0001^*^	0.2526	0.1692	0.4216	0.6972	0.0054^*^	0.1646	0.0129^*^	0.6501	0.1834	0.1026	0.0070^*^		
	p [f vs. s]										0.0495^*^		0.1996			
9	a	29.57	26.74	36.42	33.61	31.25	29.75	38.67	20.55	16.78	21.77	31.71	27.74	29.58	29.15 ± 6.35	22.20 ± 1.24
	m	53.48	56.98	49.01	56.30	54.17	54.43	50.67	67.81	59.44	64.25	55.40	57.66	59.15	56.28 ± 5.04	66.77 ± 2.28
	t	16.96	16.28	14.57	10.08	14.58	15.82	10.67	11.64	23.78	13.98	12.89	14.60	11.27	14.57 ± 3.87	11.03 ± 1.45
	p [T vs. C]	0.0160^*^	0.0879	0.0006^*^	0.0225^*^	0.0272^*^	0.0313^*^	0.0003^*^	0.9173	0.0002^*^	0.6400	0.0423^*^	0.1510	0.1894		
	p [f vs. s]										0.0992		0.5667			
10	a	29.82	28.72	22.30	22.73	23.61	37.06	45.45	12.34	21.79	18.75	28.12	22.22	23.13	26.29 ± 8.83	26.79 ± 2.16
	m	55.50	56.38	54.68	66.23	58.33	55.24	36.36	71.72	58.97	61.46	56.7	59.48	58.13	57.37 ± 8.66	60.47 ± 2.41
	t	14.68	14.89	23.02	11.04	18.06	7.69	18.18	13.10	19.23	19.79	15.18	18.30	18.75	16.09 ± 4.32	12.74 ± 1.75
	p [T vs. C]	0.5926	0.6775	0.0082^*^	0.4989	0.2625	0.0409^*^	< 0.0001^*^	0.0064^*^	0.1178	0.0422^*^	0.6810	0.1997	0.1804		
	p [f vs. s]										0.0854		0.9618			
11	a	24.82	24.55	30.91	23.53	27.97	25.00	30.88	34.21	21.74	23.86	25.87	25.81	29.88	27.21 ± 3.83	23.01 ± 1.93
	m	56.20	70.00	53.94	54.71	54.55	60.00	52.21	51.32	61.74	49.62	54.44	55.38	49.39	56.31 ± 5.77	58.9 ± 1.76
	t	18.98	5.45	15.15	21.76	17.48	15.00	16.91	14.47	16.52	26.52	19.69	18.82	20.73	16.48 ± 4.34	18.1 ± 2.83
	p [T vs. C]	0.8569	0.0040^*^	0.1644	0.5944	0.4937	0.6964	0.1712	0.0279^*^	0.8416	0.0669	0.6594	0.7484	0.1375		
	p [f vs. s]										0.2258		0.4827			
18	a	35.86	22.73	25.71	24.83	23.94	24.44	39.49	22.89	21.83	23.58	28.03	15.75	36.81	26.75 ± 7.35	22.75 ± 0.94
	m	46.21	54.55	56.43	57.93	47.18	57.78	47.13	55.42	62.68	60.16	51.51	58.90	48.47	53.88 ± 5.68	57.47 ± 1.55
	t	17.93	22.73	17.86	17.24	28.87	17.78	13.38	21.69	15.49	16.26	20.46	25.34	14.72	19.37 ± 4.76	19.79 ± 1.43
	p [T vs. C]	0.0070^*^	0.7464	0.7437	0.7701	0.0480^*^	0.8473	0.0003^*^	0.8797	0.4858	0.6751	0.3933	0.1536	0.0033^*^		
	p [f vs. s]										0.2208		< 0.0001^*^			
X	a	50.00	27.50	34.62	51.80	36.96	44.00	47.33	39.29	41.5	43.02	23.97	21.01	40.44	39.30 ± 9.30	40.77 ± 2.74
	m	44.40	65.00	56.92	44.88	51.45	52.71	43.51	52.68	56.13	45.35	68.49	69.57	47.79	53.98 ± 8.29	53.16 ± 2.40
	t	5.60	7.50	8.46	3.32	11.59	3.29	9.16	8.04	2.37	11.63	7.54	9.42	11.76	7.79 ± 3.28	6.07 ± 2.01
	p [T vs. C]	0.1678	0.0261^*^	0.3439	0.0633	0.0662	0.4647	0.1119	0.7056	0.2961	0.0415^*^	0.0029^*^	0.0003^*^	0.0529		
	p [f vs. s]										< 0.0001^*^		< 0.0001^*^			
Y	a	40.31	20.75	21.77	46.17	27.74	46.38	37.59	31.40	37.50	32.82	21.71	18.84	34.97	32.72 ± 9.79	32.48 ± 8.52
	m	52.04	60.38	66.13	49.88	56.20	48.42	50.83	61.35	57.42	51.15	61.24	68.84	58.04	56.75 ± 6.65	56.87 ± 7.08
	t	7.65	18.87	12.10	3.95	16.0645	5.20	11.58	7.25	5.08	16.03	17.05	12.32	6.99	10.53 ± 4.82	10.66 ± 3.92
	p [T vs. C]	0.2072	0.0046^*^	0.0730	0.0044^*^	0.1795	0.0067^*^	0.4665	0.4772	0.1571	0.1936	0.0209^*^	0.0142^*^	0.4775		
	p [f vs. s]										0.0246^*^		0.0012^*^			

Frequency (%) of centromeres was estimated in three regions of sperm cell nucleus: ‘a’ – near the acrosome, ‘m’ – its middle part and ‘t’ – near the tail of spermatozoa (according to the scheme presented in Fig. 1a). All RCT results were compared to the mean Control value obtained for 8 healthy donors (*p* values: T vs. C). Additionally, comparison between fathers and sons in familial RCT cases was performed: 46,XY,t(4;5)(p15.1;p12) (T10 vs. T11) and 46,XY,t(7;10)(p21.1;q26.13) (T12 vs. T13) (*p* values: f vs. s). One hundred and-fifty of centromeres were counted for each male and per each chromosome. Values *p* < 0.05 indicated statistically significant differences (two-tailed χ^2^ test) and were marked with ‘*’ in the Table

Analysis of the linear positioning revealed that the centromeres of all evaluated chromosomes in control males were preferentially localized in the middle part of the sperm nucleus (‘m’; 53.16–66.85%) (Table 2). Near the acrosome (‘a’), the frequency of spermatozoa varied from 15.46–40.77%, with the most acrosomal position for centromeres of chromosomes X and Y (40.77 and 32.48%, respectively). Localization near the sperm tail (‘t’) exhibited values from 6.07–24.42% and was preferentially exhibited by centromeres of chromosomes 4 and 8 (24.42 and 24.22%, respectively) (Table 2).

When considering the RCT group, the centromeres of all the analysed chromosomes revealed a preference for ‘middle’ localization (52.38–57.37%). The mean frequency in the ‘a’ area varied from 21.59–39.30%, while near the tail (‘t), it ranged from 7.79–23.55% (Table 2). A lower value noticed in the ‘m’ area (vs. control) was linked to a higher value in the ‘a’ area, indicating that in the RCT group, centromeres were repositioned towards the acrosome. The most acrosomal position was found for centromeres of chromosomes X and Y (39.30 and 32.72%, respectively), similarly to the controls. A preferential ‘tail’-position for chromosomes 4 and 8 was observed (22.50 and 23.55%, respectively; Table 2).

In both RCT families, there were no significant differences in the localization of chromosomes 7, 9 and 11 in comparison to the mean control values (Table 2, Fig. 2). Additionally, no repositioning was observed for the chromosomes: 18 in the t(4;5) family and 10 in the t(7;10) case. Statistically significant differences (*p* < 0.05), simultaneously between fathers and sons vs. mean control values, were found for the centromere of chromosome 4, which was shifted towards the acrosome area (similarly in both families), and for chromosome X in the t(4;5) family. Single changes (for father or son vs. control) in centromere localization were noted in the t(4;5) family for chromosomes 10 and Y and in the t(7;10) family for chromosomes 8, 18, X and Y (Table 2, Fig. 2). When considering differences in father vs. son, significant differences (*p* < 0.05) were observed in both families for sex chromosomes. Further repositions were also found for chromosome 8 in the t(4;5) family and for chromosome 18 in the t(7;10) case (Table 2, Fig. 2).

### Radial positioning

**Fig. 3:**
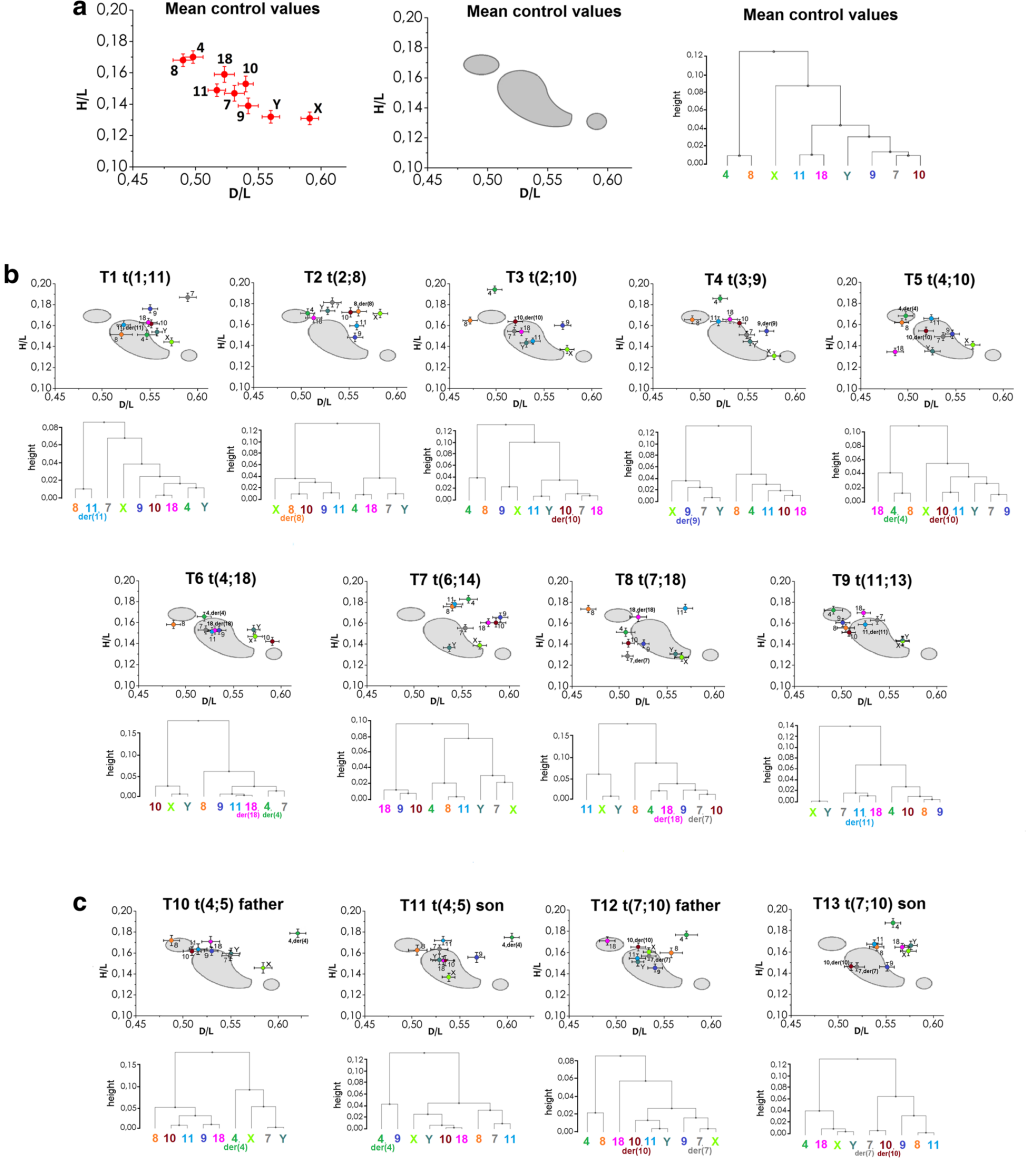
Radial positioning of the analysed centromeres (4, 7, 8, 9, 10, 11, 18, X, Y) in the sperm cell nucleus, according to the values D/L ± SE (OX axis) and H/L ± SE (OY axis) from Table 3. **a** The mean control values obtained for eight control donors: positioning of particular centromeres (left), three areas of the chromocenter (middle; grey areas), and hierarchical Ward analysis representing the clustering of centromeres (right). **b** Positioning results for nine RCT carriers (T1-T9), including a comparison with the control chromocenter areas (grey). **c** Positioning results for familial RCT cases (T10 and T11 – t(4;5); T11 and T12 – t(7;10))

**Fig. 4:**
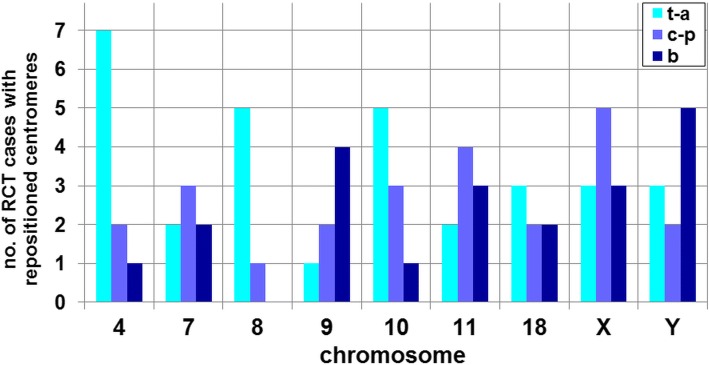
Number of RCT cases with radial repositioning of particular chromosomes, including changes in the ‘tail-acrosome’ direction (t-a), ‘centre-periphery’ (c-p) or both (b)

**Fig. 5:**
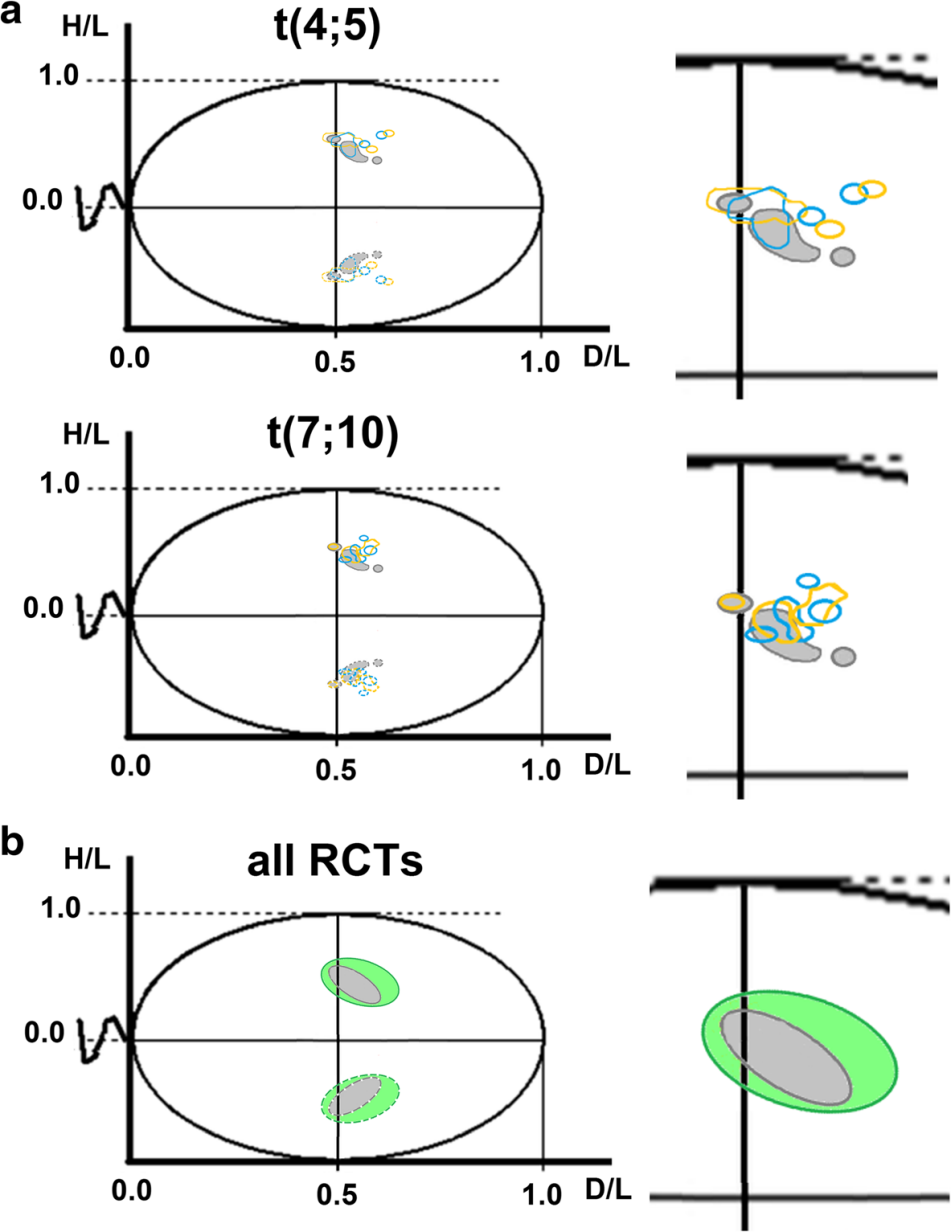
Schematic representation of the sperm nuclear area occupied by chromocenters with the chromosome centromeres under study (4, 7, 8, 9, 10, 11, 18, X, Y). Solid lines show the observed chromocenter areas; dotted lines show their mirror images. The extent of all areas is depicted by the mean values D/L ± SE and H/L ± SE presented in Table 3 and Fig. 3 and in Additional file 1: Figure S1. **a** The results obtained for familial RCT cases with chromocenter marked data from fathers (yellow), sons (blue), and the mean control (grey). **b** The results obtained for all evaluated RCT carriers (T1-T13; green colour) vs. all controls (grey colour)

**Fig. 6:**
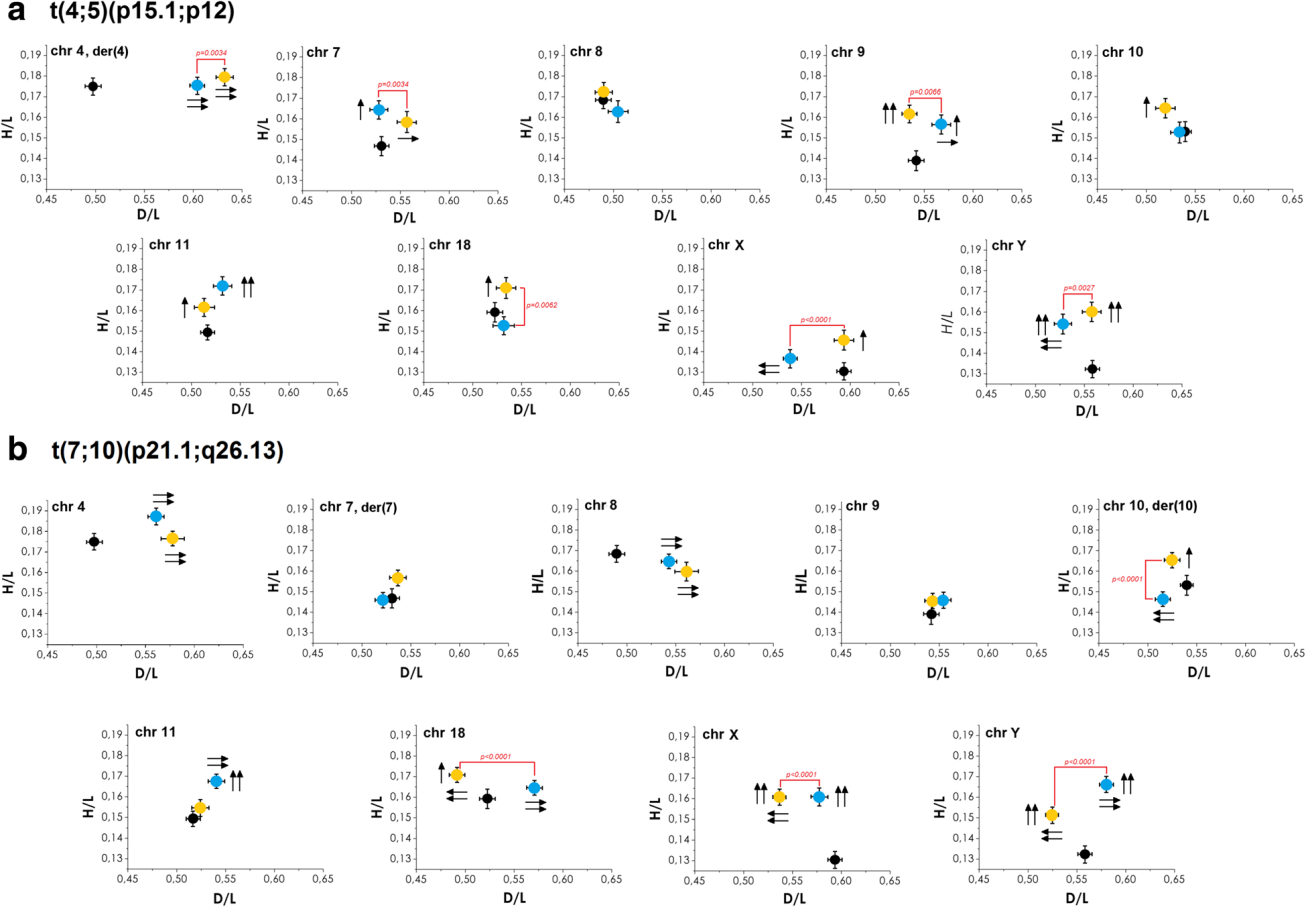
Radial positioning of chromosome centromeres 4, 7, 8, 9, 10, 11, 18, X and Y in two familial RCT cases (T10 and T11; T12 and T13). **a** 46,XY,t(4;5)(p15.1;p12). **b** 46,XY,t(7;10)(p21.2;q26.13). Statistically significant differences between father (yellow) and son (blue) are marked in red. Spots that differ significantly from the mean control value (black) are indicated by arrows: double arrow for *p* ≤ 0.0001, single arrow for 0.0001 < *p* ≤ 0.01. Arrows also indicate the direction of the observed shift/repositioning of centromeres. Bars show standard errors (SE)

The radial positioning results are presented in Tables 3, 4 and 5 and in Figs. 3, 4, 5 and 6.

**Table 3:** Radial positioning of centromeres of chromosomes 4, 7, 8, 9, 10, 11, 18, X and Y in spermatozoa of 13 reciprocal translocation carriers (RCT)

Chromosome		T1	T2	T3	T4	T5	T6	T7	T8	T9	T10	T11	T12	T13	Control mean value [*n* = 8]
		t(1;11)	t(2;8)	t(2;10)	t(3;9)	t(4;10)	t(4;18)	t(6;14)	t(7;18)	t(11;13)	t(4;5) father	t(4;5) son	t(7;10) father	t(7;10) son	
4	D/L	0.547^↑↑^	0.507	0.498	0.521^↑^	0.505	0.519^↑^	0.557^↑↑^	0.500	0.491	0.632^↑↑b^	0.604^↑↑b^	0.578^↑↑^	0.561^↑↑^	0.498
	se	0.015	0.008	0.008	0.008	0.008	0.008	0.009	0.001	0,010	0.009	0.008	0,012	0.008	0.008
	H/L	0.151^↓^	0.171	0.194^↑↑^	0.186	0.169	0.166	0.183	0.153^↓↓^	0.172	0.179	0.175	0.177	0.187	0.175
	se	0.005	0.004	0.003	0.003	0.004	0.004	0.004	0.004	0.004	0.004	0.004	0.004	0.004	0.004
7	D/L	0.590^↑↑^	0.533	0.518	0.549	0.539	0.521	0.554^↑^	0.502^↓^	0.538	0.556^↑b^	0.528^b^	0.537	0.522	0.531
	se	0.009	0.009	0.007	0.008	0.009	0.008	0.008	0.006	0.008	0,010	0.009	0.008	0.008	0.008
	H/L	0.187^↑↑^	0.181^↑↑^	0.155	0.151	0.149	0.153	0.155	0.129^↓↓^	0.163^↑^	0.158	0.164^↑^	0.157	0.146	0.147
	se	0.004	0.004	0.004	0.004	0.004	0.004	0.004	0.004	0.004	0.005	0.004	0.004	0.004	0.005
8	D/L	0.520	0.561^↑↑^	0.472	0.492	0.495	0.487	0.540^↑↑^	0.458^↓^	0.504	0.490	0.505	0.561^↑↑^	0.543^↑↑^	0.490
	se	0.012	0.009	0.008	0.009	0.008	0.007	0,010	0.009	0.009	0.009	0,010	0,012	0.008	0.008
	H/L	0.151^↓^	0.173	0.165	0.166	0.162	0.158	0.176	0.174	0.156	0.172	0.163	0.160	0.165	0.168
	se	0.004	0.004	0.003	0.004	0.004	0.004	0.004	0.004	0.003	0.005	0.005	0.005	0.004	0.004
9	D/L	0.550	0.557	0.569^↑↑^	0.570^↑↑^	0.549	0.535	0.591^↑↑^	0.520	0.501^↓↓^	0.535^b^	0.567^↑b^	0.543	0.554	0.542
	se	0.008	0.008	0.009	0.008	0.008	0.007	0.008	0.006	0.007	0.008	0.009	0.008	0.008	0.008
	H/L	0.176^↑↑^	0.148	0.160^↑↑^	0.155	0.151	0.153	0.166^↑↑^	0.140	0.160^↑↑^	0.162^↑↑^	0.156^↑^	0.146	0.146	0.139
	se	0.004	0.004	0.004	0.004	0.004	0.004	0.004	0.004	0.004	0.004	0.005	0.004	0.004	0.005
10	D/L	0.552	0.552	0.519^↓^	0.541	0.521	0.591^↑↑^	0.586^↑↑^	0.503^↓↓^	0.508^↓↓^	0.520	0.534	0.525	0.515^↓↓^	0.540
	se	0.009	0.009	0.008	0.006	0.008	0.007	0.011	0.007	0.007	0.010	0.010	0.008	0.008	0.006
	H/L	0.162	0.172^↑↑^	0.164^↑^	0.162	0.154	0.142	0.160	0.141	0.151	0.164^↑^	0.153	0.165^↑a^	0.146^a^	0.153
	se	0.004	0.005	0.004	0.004	0.004	0.004	0.004	0.004	0.004	0.005	0.005	0.004	0.004	0.005
11	D/L	0.522	0.559^↑↑^	0.538^↑^	0.519	0.527	0.529	0.543^↑^	0.567^↑↑^	0.525	0.513	0.532	0.524	0.541^↑↑^	0.517
	se	0.007	0.007	0.008	0.008	0.008	0.008	0.009	0.008	0.008	0.010	0.009	0.009	0.008	0.007
	H/L	0.161	0.159	0.145	0.164^↑↑^	0.166^↑↑^	0.151	0.178^↑↑^	0.174^↑↑^	0.159	0.162^↑^	0.172^↑↑^	0.155	0.168^↑↑^	0.149
	se	0.004	0.004	0.003	0.004	0.004	0.004	0.004	0.004	0.004	0.004	0.004	0.004	0.004	0.004
18	D/L	0.549^↑^	0.513	0.526	0.531	0.487^↓↓^	0.531	0.578^↑↑^	0.515	0.523	0.534	0.532	0.492^↓↓a^	0.571^↑↑a^	0.523
	se	0.009	0.009	0.008	0.008	0.009	0.007	0.008	0.010	0.007	0.010	0.011	0.008	0.008	0.008
	H/L	0.163	0.166	0.154	0.166	0.134^↓↓^	0.153	0.160	0.166	0.170^↑^	0.171^↑b^	0.153^b^	0.171^↑^	0.165	0.159
	se	0.004	0.004	0.004	0.004	0.004	0.004	0.004	0.004	0.003	0.005	0.004	0.004	0.004	0.005
X	D/L	0.573	0.583	0.574	0.578	0.572^↓^	0.573	0.569^↓↓^	0.564^↓↓^	0.564^↓↓^	0.593^a^	0.538^↓↓a^	0.537^↓↓a^	0.578^a^	0.593
	se	0.008	0.007	0.008	0.007	0.008	0.008	0.007	0.008	0.007	0.010	0.007	0.007	0.009	0.007
	H/L	0.144^↑^	0.171^↑↑^	0.137	0.131	0.141^↑^	0.147^↑↑^	0.139	0.128	0.143^↑^	0.146^↑^	0.137	0.161^↑↑^	0.161^↑↑^	0.130
	se	0.004	0.004	0.004	0.004	0.004	0.004	0.004	0.004	0.004	0.005	0.004	0.004	0.004	0.004
Y	D/L	0.558	0.528^↓↓^	0.531^↓↓^	0.553	0.528^↓↓^	0.572^↑↑^	0.537^↓↓^	0.557	0.564	0.557^b^	0.528^↓↓b^	0.525^↓↓a^	0.580^↑↑a^	0.558
	se	0.006	0.008	0.007	0.008	0.008	0.007	0.006	0.006	0.007	0,010	0.009	0.006	0.007	0.007
	H/L	0.154^↑↑^	0.173^↑↑^	0.143	0.145	0.135	0.153^↑↑^	0.137	0.131	0.143	0.160^↑↑^	0.154^↑↑^	0.151^↑↑^	0.166^↑↑^	0.132
	se	0.004	0.004	0.004	0.004	0.004	0.004	0.004	0.003	0.004	0.005	0.005	0.004	0.004	0.004

Carriers: T10-T13 represent two familial cases of fathers and sons: 46,XY,t(4;5)(p15.1;p12) (T10, T11) and 46,XY,t(7;10)(p21.1;q26.13) (T12, T13). All results were compared to mean Control value obtained for 8 healthy donors. One hundred and-fifty of centromeres were counted for each male and per each chromosome. Values *p* < 0.01 indicated statistically significant differences (one-way ANOVA) and were marked with proper asterisks

Asterisks used in the Table mark the power of significance

when compared to mean Control value

*p* ≤ 0.0001 (very high statistical significance)

^↑↑^ centromere localized closer to the acrosome

^↓↓^ centromere localized closer to the basal part

*p* > 0.0001-*p* ≤ 0.01(high statistical significance)

^↑^ centromere localized closer to the acrosome

^↓^ centromere localized closer to the basal part;

between father and son

^a^ *p* ≤ 0.0001 (very high statistical significance)

^b^ 0.0001 < *p* ≤ 0.01 (high statistical significance)

**Table 4:** Summary of radial positioning – chromosomes with changed localization of centromeres in particular RCT cases, with reference to mean control positioning values

RCT		CHROMOSOME			
		Direction of repositioning			
		Tail-acrosome	Center-periphery	Both	None
T1	t(1;11)	18	8, 9, X, Y	4, 7	10, 11/der(11)
T2	t(2;8)	8/der(8), 11	7, 10, X	Y	4, 9, 18
T3	t(2;10)	11, Y	4	9, 10/der(10)	7, 8, 18, X
T4	t(3;9)	4, 9/der(9)	11	–	7, 8, 10, 18, X, Y
T5	t(4;10)	Y	11	18, X	4/der(4), 7, 8, 9, 10/der(10)
T6	t(4;18)	4/der(4), 10	X	Y	7, 8, 9, 11, 18/der(18)
T7	t(6;14)	4, 7, 8, 10, 18, X, Y	–	9, 11	–
T8	t(7;18)	8, 10, X	4	7/der(7), 11	9, 18/der(18), Y
T9	t(11;13)	10	7, 18	9, X	4, 8, 11/der(11), Y
Familial cases:					
T10	t(4;5) father	4/der(4), 7	9, 10, 11, 18, X, Y	–	8
T11	t(4;5) son	4/der(4), X	7, 11	9, Y	8, 10, 18
T12	t(7;10) father	4, 8	10/der(10)	18, X, Y	7/der(7), 9, 11
T13	t(7;10) son	4, 8, 10/der(10), 18	X	11, Y	7/der(7), 9

**Table 5:** Summary of differences observed in radial positioning observed in familial RCT cases. Results presented in details in Table 3 and Fig. 4. ‘+’ means the difference in topology of particular centromere in cell nucleus of spermatozoa between father and son (*p* < 0.01)

RCT		Chromosome								
		4	7	8	9	10	11	18	X	Y
T10 T11	t(4;5) father t(4;5) son	+	+		+			+	+	+
Direction of repositioning		S-c	S-c		S-a			S-c	S-c	S-c
T12 T13	t(7;10) father t(7;10) son					+		+	+	+
Direction of repositioning						F-p		F-c	F-c	F-c

S-c: positions of centromeres localized more central in spermatozoa of son when compared to father

S-a: positions of centromeres localized more acrosomal in spermatozoa of son when compared to father

F-p: positions of centromeres localized more peripheral in spermatozoa of father when compared to son

F-c: positions of centromeres localized more central in spermatozoa of father when compared to son

The results obtained in control group are presented in Table 3 (mean values) and Fig. 3a. These results showed that based on (i) the ‘tail-acrosome’ criterion, centromeres were localized in the following order: 8, 4, 11, 18, 7, 10, 9, Y and X; (ii) ‘centre-periphery’ criterion, the most peripheral position was assumed by chromosomes 4 and 8, in contrast to chromosomes X and Y, which were located the most centrally, deep in the sperm nucleus. Hierarchical clustering showed that some chromosomes were localized pair-wise (4 and 8, 11 and 18), in a group (Y, 7, 9 and 10) or remaining single (X). Three control chromocenter areas are depicted (Fig. 3a, right panel).

The radial positioning results in the RCT carrier group are shown in Tables 3 and 4 and in Figs. 3bc, 4 and 5. Radial positioning analysis in the RCT group revealed several changes in the chromosome order within the sperm nucleus. In comparison to the control region (grey background areas in Fig. 3), repositioning of the centromeres of particular chromosomes was clearly observed (Fig. 3). Additionally, hierarchical clustering showed differences in the grouping of centromeres in individual RCT carriers, presenting potentially 2–4 clusters – chromocenters (Fig. 3bc). When considering the ‘stability’ of centromere positioning, this point finding can be investigated both from chromosomal and from individual RCT carrier aspects. Thus, when examining the chromosomal issue, the most statistically ‘stable’ centromere position was found for chromosomes 8 (7/13 RCT cases), 7, 9 and 18 (6/13) (*p* ≥ 0.01; Tables 3 and 4, Fig. 4). In contrast, when analysing chromosomes that were mostly repositioned, sex chromosome localization was changed in 10/13 RCT (X) and 11/13 (Y) cases, followed by chromosome 4 (10/13) (*p* < 0.01; Table 4, Fig. 4). Interestingly, a small number of repositioned chromosomes (≤ 4; *p* < 0.01) was observed only in three RCT cases (T4, T5, T6). In contrast, the most ‘unstable’ RCT was T7 t(6;14), with a statistically changed localization in all nine chromosomes evaluated (*p* < 0.01; Table 4).

The detailed results of the topology examination of familial cases is presented in Figs. 3c and 6 and Tables 3 and 5. In both RCT families, the strongest relocation (vs. control; *p* < 0.01) was found for chromosome 4, with its centromere visibly shifted to the acrosomal area. Chromosomes X and Y were clearly repositioned towards the periphery of the sperm nucleus (*p* < 0.01). In contrast, no repositioning vs. control (*p* ≥ 0.01) was noted for chromosome 8 in the t(4;5) family, followed by chromosomes 7 and 9 in the case of t(7;10).

When considering the t(4;5) family (Fig. 6a), the centromeres of chromosomes 4, 9, 11 and Y were similarly shifted for father and son simultaneously when compared to the mean control results: chromosome 4 was repositioned towards the acrosome (*p* < 0.0001), while chromosomes 9, 11 and Y were repositioned to the nuclear periphery (*p* < 0.01). When considering chromosomal positioning between father and son, no dissimilarities were found for the centromeres of chromosomes 8, 10 and 11 (*p* ≥ 0.01), while statistically significant differences were observed for the centromeres of chromosomes 4, 7, 9, 18, X and Y (*p* < 0.01).

The results obtained for the second familial RCT case t(7;10) were in agreement with these observations (Fig. 6b). Namely, the centromeres of chromosomes 4, 8, X and Y were repositioned in a similar way in father and son when compared to the control: chromosomes 4 and 8 were shifted towards the acrosomal area, while X and Y were repositioned to the nuclear periphery (*p* < 0.0001). No statistically significant differences in positioning (father vs. son; *p* ≥ 0.01) were found for the centromeres of chromosomes 4, 7, 8, 9 and 11, in contrast to the other chromosomes with visible repositions in the father’s gametes into the sperm periphery (chromosome 10) or sperm tail region (18, X and Y) (*p* < 0.0001).

The number of chromocenter areas was similar between fathers and sons (3 or 4) and when compared to the control (3 areas) (Figs. 3 and 5a). These areas contained different configurations of chromosomes as follows: single, pair-wise or grouped (Fig. 3c; hierarchical clustering). When considering the total nuclear region occupied by the nine analysed chromosomes, the nuclear territory was clearly approximately twice as wide in RCT carriers compared with the control (Fig. 5b).

### Chromosome characteristics

Measurement of proper parts/elements of chromosomes involved in RCTs revealed that various fragment lengths of chromosomal arms were involved in translocations (TS: 3.01–91.47% of the whole arm; Additional file 2: Table S1). In 6 cases of 5 RCTs (T4, T5, T6, T9, T12/T13), the percentage ratio of TS was below 50% in both chromosomes simultaneously. These findings indicated that no more than half of a proper chromosomal arm was translocated into its rearrangement partner. Six breakpoint positions were located near the centromere and six in the subtelomeric region. In 3 RCTs (T1, T9, T12/T13), both p and q chromosomal arms were involved in the rearrangement. The presence of an acrocentric chromosome was noted in 2 RCT cases (T7, T9).

To clarify the order in the present study, we have provided a short summary of the data concerning hyperhaploidy, the genetic imbalance ratio, and chromatin integrity for the spermatozoa of the evaluated RCT carriers in Table 1, which have been were previously published [45, 47–49, 51]. Because of the individual characteristics of each RCT, no mean values were estimated, and the obtained results were compared only to mean control values.

### Hyperhaploidy in spermatozoa

Analysis of the frequency of spermatozoa with an additional chromosome showed that among nine analysed chromosomes, hyperhaploidy of XY was observed in 11 of 13 RCT carriers (range: 0.16–0.73%), followed by chromosomes 18 (7/11; 0.22–0.64%), 10 (5/9; 0.04–0.31%), and 8 (6/12; 0.06–0.60%) (Table 1). Hyperhaploidies of other chromosomes were also noticed in less than 50% of RCT cases. In two RCT carriers (T4, T7), an interchromosomal effect was observed (hyperhaploidies of all analysed chromosomes were increased compared with control results). In the familial case of t(4;5), an increased hyperhaploidy ratio was observed for chromosomes 18 and XY in both family members. Additionally, in the spermatozoa of the father, hyperhaploidies of chromosomes 10, XX and YY were also noticed. In the second family t(7;10), XY hyperhaploidy was found in both males, followed by chromosomes 4 and 8 in the father’s spermatozoa and chromosome 9 in the sperm cells of the son (Table 1).

### Meiotic segregation pattern

The genetic imbalance ratio in the spermatozoa of the studied RCT carriers varied from 37.6 to 65.7%. In both familial cases, the results characterizing father and son showed similar values (t(4;5) father 65.2% vs. son 65.6%; t(7;10) father 37.6% vs. son 44.4%).

### Chromatin integrity

Sperm chromatin integrity was analysed using aniline blue (AB) staining for determination of the frequency of spermatozoa with deprotaminated chromatin, and the TUNEL assay to check the frequency of sperm cells with fragmented DNA. The frequency of sperm cells with deprotaminated chromatin ranged from 4.6 to 66.3% in the RCT group (Table 1). Compared with the controls, a higher level of deprotaminated sperm chromatin was found in 4 of 10 RCT cases, including 3 carriers, with frequencies of spermatozoa with deprotaminated chromatin > 35% (T3–66.3%, T5–36.5%, T8–45.6%). In the familial case of t(4;5), the frequency of deprotaminated spermatozoa was 3-fold higher in the son vs. father (13.7% vs. 4.6%). In the familial case of t(7;10), only the son’s sperm cells were analysed (insufficient material from the father). The TUNEL assay results revealed a frequency of spermatozoa with fragmented DNA of 5.6–35.8% (Table 1). Additionally, 6 of 11 RCT carriers showed a higher frequency of spermatozoa with fragmented DNA compared with the control group, including two males with values > 30% (T3, T6), four between 15 and 30% (T2, T7, T9, T12), and five below 10% (T1, T4, T5, T8, T13). In the familial case of t(7;10), the frequency of spermatozoa with fragmented DNA was 4.6-fold higher in father vs. son (25.9% vs. 5.6%). Because of the insufficient quantity of material from the t(4;5) family, the TUNEL assay was not performed in this case.

## Discussion

This is the first study to examine the topology of the chromosomes in the sperm cell nucleus of male carriers with the same balanced reciprocal chromosome translocation in two familial cases of fathers and sons. Additionally, chromosome positioning analysis was performed in a group of other RCT carriers to underline the superior role of the presence of an abnormal karyotype vs. sperm chromatin integrity components. We also assessed whether the sperm nuclear order might be diversified in relation to the frequency of genetically unbalanced gametes. To our knowledge, this is also the biggest study conducted to date concerning sperm nuclear order alterations in males with an abnormal karyotype (11 RCTs of 13 RCT carriers; 9 chromosomes).

Our results confirmed the defined and stable localization of centromeres in healthy control males. Our findings were consistent with previously published data showing the preferential positioning of particular chromosome centromeres within the human sperm cell nucleus [6, 21, 22, 24, 52]. We showed that among the nine analysed chromosomes, the most central/deep and acrosome-close localization was assumed by chromosomes X and Y, in contrast to chromosomes 4 and 8, which were positioned the most peripherally and close to the sperm tail region. Additionally, three control chromocenter areas were observed.

In the RCT group analysed herein, we observed the greatest instability of localization among autosomes for chromosome 4 – its position was changed in 10/13 RCT cases. At present, we can hypothesize that the high mobility of chromosome 4 may originate from its large size, resulting in substantial surface exposure to mechanical forces during a topological reorganization event. Studies performed in different tissues and organisms have clearly shown that in the nuclear ordering of chromosomes, a critical role may be played by mechanisms physically governing genomic organization [53, 54]. Other authors have previously suggested that the size of a chromosome may also be important in its proper positioning in human spermatozoa [17, 19, 20]. Additionally, in most RCT males analysed in this study, repositioning of sex chromosomes was observed (X – 10/13; Y – 11/13), clearly suggesting their vulnerability to repositioning. This finding also strongly confirms previous data showing that the repositioning of sex chromosomes towards the sperm nuclear periphery accompanies disturbances in spermatogenesis in males with reproductive failure [21–25, 27, 28, 55]. In control spermatozoa, preferential X chromosome localization was observed in the acrosomal area, which is the first region that interacts with the ooplasm during fertilization [6, 13, 22–24, 52, 55, 56]. It was therefore suggested that such a chromosome X position may play a functionally important role in the reorganization of the paternal genome and inactivation of X chromosome [15, 57]. Specific apical positioning of the centromere of the X chromosome has also been documented in spermatozoa rinsed away from the oocyte surface after a sperm penetration assay (SPA) into hamster egg, demonstrating a lack of chromosome X presence near the tail area of the sperm nucleus [58]. Simultaneously, the positioning of chromosome X deep inside the sperm nucleus seems to be protective for its genetic content [13, 55, 59].

When evaluating two familial cases, repositioning of the chromosomes occurred in a similar way when compared to the control results, even though some chromosomal differences between father and son were observed. An example is that the centromeres of chromosomes 4 and 8 were shifted towards the acrosome area in familial cases, while in controls they colocalized and assumed a position in the middle part of the sperm nucleus. Moreover, sex chromosomes were also shifted similarly in fathers and sons towards the periphery of the sperm nucleus from the fixed deep central region in controls. The observation that repositioning of particular chromosomes was similar in a father and son allowed us to suggest that substantial RCT with definite chromosome involvement might influence the nuclear order in a determined fashion.

In this study, we also observed that 3 main geometrical features of chromosomes involved in RCT may be essential for a scale of chromosome repositioning in the human sperm cell nucleus. First, we found an important role of the length of the translocated segment (TS) of the chromosomal arms involved in a particular rearrangement (see Additional file 2: Table S1). We observed the smallest number of repositioned chromosomes (≤ 4; *p* < 0.01) in only three RCT cases (T4, T5, T6) – in all of them, the ratio of exchanged genetic material was less than 50% of the arms of both involved chromosomes; thus, the final asymmetry between the chromosome and its derivative remained limited and stable (see Additional file 1: Figure S1, Additional file 2: Table S1). Second, we can hypothesize that the engagement of opposite arms in RCT may destabilize the nuclear order of sperm chromosomes, even if the TS value was < 50%. This phenomenon has been observed in T12/T13 cases with the involvement of both chromosomal arms (p and q). Third, the presence of an acrocentric chromosome seems to be meaningful and enhance destabilization of the chromosome position. Its role was observed in the case of T9, in which more than half of the chromosomes evaluated were repositioned, even if TS was < 50% and simultaneous involvement of p and q was found. The influence of an acrocentric chromosome was also observed in the case of the most ‘unstable’ RCT – T7, with altered positions of all nine chromosomes evaluated. Thus, we can clearly assume that 3 geometrical features, distance of the breakpoint to the centromere (TS < 50%), rearrangement between opposite chromosomal arms, and involvement of the acrocentric chromosome, may successfully intensify repositioning of the chromosomes in the human sperm cell nucleus.

The chromosomal geometry seems to also be prominent over the presence of an additional chromosome – hyperhaploidy. Of course, the influence of hyperhaploidy, as another chromosomal factor, on the positioning of chromosomes within the sperm nucleus remains unquestionable [22–24, 60]. Our observation from RCT cases with increased hyperhaploidy of a particular chromosome showed that the topology of this chromosome was also altered in such cases (in 6/13 RCTs). Interesting findings were revealed when analysing the number of hyperhaploid chromosomes in familial RCT cases, in which we found that a higher level of hyperhaploidy of X and Y chromosomes resulted in a greater number of repositioned chromosomes. Sex chromosome hyperhaploidies appear to influence the number of repositions in the case of the same chromosomal rearrangements (karyotype). Thus, the presence of any hyperhaploidy may in fact disturb the nuclear organization. Taken together, these observations seem to confirm previously published data documenting an altered chromosome topology in hyperhaploidic spermatozoa [22–24, 60]. The most visible repositionings were observed for the T7 case with a suggested interchromosomal effect (ICE) and repositioning of all the studied chromosomes. It seems logical to suggest that in cases with undoubtedly documented ICE, the presence of an additional chromosome within the nuclear space may be more important than the particular karyotype of the analysed case per se. This hypothesis is further supported by data published with supernumerary marker chromosome (sSMC) carriers, in which the presence of sSMC – which is in fact also a hyperhaploidy – was followed by documented ICE in spermatozoa and was accompanied by altered chromosomal positions [19, 23, 28]. However, in the present study, ICE was also observed in the T4 case, with the lowest number of repositioned chromosomes (3/9) among the whole RCT group (Table 4). These findings suggest the presence of some other factor (than hyperhaploidy by itself) that influences chromosome repositioning. We can hypothesize/claim that the difference between the two cases resulted from the geometrical features of the chromosomes involved in RCT (discussed in the previous section). Thus, we suggest that between the two chromosomal factors (geometry vs. hyperhaploidy), geometrical features seem to be superior.

The next point addressed in this study concerned whether nuclear topology in sperm might differ in relation to the frequency of genetically unbalanced gametes. In our group of RCTs, the frequencies of genetically unbalanced spermatozoa varied from 37.6 to 65.7% (Table 1). We did not identify a direct link between the number of repositioned chromosomes and the genetic imbalance ratio, either in the whole RCT group or in familial cases. RCT cases with the most reshuffled chromosomes (≥7/9) manifested both a high frequency of genetically unbalanced gametes (approx. 60%; T1, T10) and a medium value of genetic imbalance (approx. 45%; T7, T13). Similar observations were performed in RCT cases with an almost intact nuclear order (T4, T5, T6). The lack of a clear link was also underlined by different levels of chromosome repositioning (4–9/9) in RCT cases with similar frequencies of genetically unbalanced gametes (i.e., T5 and T12, or T7, T9 and T13). Thus, we can only claim that the presence of the chromosomal rearrangement itself alters the nuclear order in spermatozoa and is rather independent from the genetic imbalance resulting from the meiotic segregation of the chromosomes involved in the rearrangement.

Our study also corroborates the tendency of centromeres to aggregate when composing chromocenters (Figs. 3 and 5) [11, 26, 52]. We found that in the whole RCT group, the number of chromocenters varied between 2 and 3 areas and was similar to the mean 3 control areas. Additionally, in familial cases, the number of chromocenter areas was similar between fathers and sons (3 or 4) and when compared to controls (3 areas) (Fig. 5a). Interestingly, the nuclear territory occupied by the analysed nine chromosomes was approximately twice as wide in the group of all RCT carriers compared with the control (Fig. 5b). This finding confirms previously published data that in males with disrupted spermatogenesis (both an abnormal karyotype or sperm chromatin integration disturbances), the number of chromocenters may be changed, with a tendency to occupy a wider area of the sperm nuclear space when compared to controls with intact spermatogenesis (Figs. 3 and 5) [22–24, 26].

In this study, we also examined the role of chromatin integrity components (sperm deprotamination and DNA fragmentation) in the sperm nuclear order of chromosomes. In our previous study [25], observations of chromosome positioning were performed in spermatozoa with fragmented DNA (TUNEL positive) from a patient with a normal karyotype (46,XY). We have suggested some link between sperm DNA damage and nuclear topology, but without a clear determination of the cause-effect relationship. In the present study investigating spermatozoa from patients with abnormal karyotypes, we did not find any link between the number of repositioned chromosomes and disturbances in sperm DNA fragmentation. In three RCT cases with a low frequency of spermatozoa with fragmented DNA (< 10%; T1, T8, T13; Table 1), the number of reshuffled chromosomes was high (≥6/9; Table 4). In contrast, cases with the highest level of sperm DNA fragmentation (> 30%; T3, T6) revealed repositioning for only half of the analysed chromosomes. In only two RCT cases with a small number of repositioned chromosomes (4/9; T4, T5) were the results of the TUNEL assay also low (< 10%). Thus, we can suggest that chromosomal rearrangement (abnormal karyotype) may play a superior role to sperm DNA damage with respect to its influence on chromosome topology. A similar conclusion may be drawn when collating the number of changed chromosomal locations with other chromatin integrity components such as chromatin deprotamination. In RCT cases with a high frequency of deprotaminated spermatozoa (> 35%; T3, T5, T8; Table 1), the number of repositioned chromosomes remained at the middle level (4–6/9; Table 4). Interestingly, in RCT cases with the most altered topology (≥7/9; T7, T10, T13), the frequencies of spermatozoa with deprotaminated chromatin did not differ from the control values. Thus, the role of chromatin integrity (both sperm DNA fragmentation and chromatin deprotamination) in the nuclear order of spermatozoal chromosomes seems to be subordinated by the presence of an abnormal karyotype. This conclusion can also be supported by data obtained for familial cases, in which despite high chromatin disintegrity, the number of repositioned chromosomes was low. Thus, we can suggest that the differences in chromosome positioning between father and son carriers of the same chromosomal rearrangement may arise rather from chromosomal but not chromatin factors. In contrast, however, chromatin integrity may play a role in some other causative effects on the chromosomal order in males with the same chromosomal rearrangement. Indeed, in the familial cases examined in this study, we observed that chromatin integrity might influence the direction of chromosomal repositioning. Namely, when high disintegration was observed, more centrally chromosomes were located (i) in the t(4;5) family, chromatin deprotamination in the son was approximately 3-fold higher than in the father, and the son’s chromosomes (4, 7, 18, X and Y) were positioned more centrally than in his father (with the exception of chromosome 9, which was localized more acrosomally); (ii) in the t(7;10) family, sperm DNA fragmentation was approximately 4.6-fold higher in the father compared with the son, and the father’s chromosomes (18, X and Y) were located more centrally than son’s chromosomes (with the exception of chromosome 10, which was observed more peripherally) (Table 4, Fig. 6). Thus, considering the chromatin integrity vs. chromosomal rearrangement, the primary role in the nuclear order is played by a chromosomal factor (number of repositioned chromosomes), followed by chromatin integrity (direction of repositions). Confirmation of this hypothesis requires further investigation in other carries with chromosomal rearrangements, not only with RCTs.

## Conclusions

In conclusion, we can suggest that a particular structural chromosome rearrangement (RCT in this study) is reflected by the individual pattern of chromosomes positioning in the sperm cell nucleus. This conclusion was shown by generally similar repositions in fathers and sons when compared with controls. Additionally, the geometric characteristics of the pair of particular chromosomes involved in RCT may determine the scale of repositioning. Furthermore, high sperm hyperhaploidy may not be neutral for nuclear order fixing/settlement. Importantly, during meiotic stages of spermatogenesis, chromosomes establish firm locations, which are then preserved in the sperm nucleus [16–18]. It has been well documented that both rearranged chromosomes and the presence of an additional chromosome can disturb meiosis via an association with the other chromosomes, leading to decreased fertility [61]. Considering all these findings together, we can claim that chromosomal factors, especially an abnormal karyotype, are superior factors influencing the topology of chromosomes in spermatozoa. The role of chromatin integrity components in the sperm nuclear order should not be neutral but can most likely be considered secondary (playing a role in the direction of repositions) when examining cases with the same karyotype (RCT).

## Additional files

Additional file 1: **Figure S1.** Schematic GTG ideograms of 11 reciprocal chromosomal translocations from 13 male carriers evaluated in the study. In each RCT, breakpoints were marked with arrows, and chromosomes from each pair were differently coloured to show the size of translocated segments (TS). (PDF 369 kb)

Additional file 2: **Table S1.** Chromosome characteristics in each of analyzed RCTs. TS – translocated fragment (from breakpoint to the end of a chromosomal arm), IS – interstitial fragment (from centromere to the breakpoint); f – father, s – son; ‘+’ – presence of a proper feature, grey colour – cases with TS values below 50% in both engaged chromosomes simultaneously. All values counted according to data from NCBI Genome Data Viewer, GRCh38.p11 (https://www.ncbi.nlm.nih.gov/genome/gdv/). (PDF 578 kb)

## Abbreviations

AB: Aniline blue
CT: Chromosome territory
DTT: Dithiotreitol
FISH: Fluorescent in situ hybridization
IC: Interchromosomal compartment
ICE: Interchromosomal effect
RCT: Reciprocal chromosome translocation
SPA: Sperm penetration assay
sSMC: Small supernumerary chromosome
TAD: Topologically associated domain
TS: Translocated segment
