# Supplementary material for: Chromosome (re)positioning in spermatozoa of fathers and sons – carriers of reciprocal chromosome translocation (RCT)
Source: BMC Med Genomics. 2019 Feb 1;12:30. doi: 10.1186/s12920-018-0470-7 (PMC6359769; doi:10.1186/s12920-018-0470-7)

Additional file 1: Figure S1. Schematic GTG ideograms of 11 reciprocal chromosomal translocations from 13 male carriers evaluated in the study. In each RCT, breakpoints were marked with arrows, and chromosomes from each pair were differently coloured to show the size of translocated segments (TS).

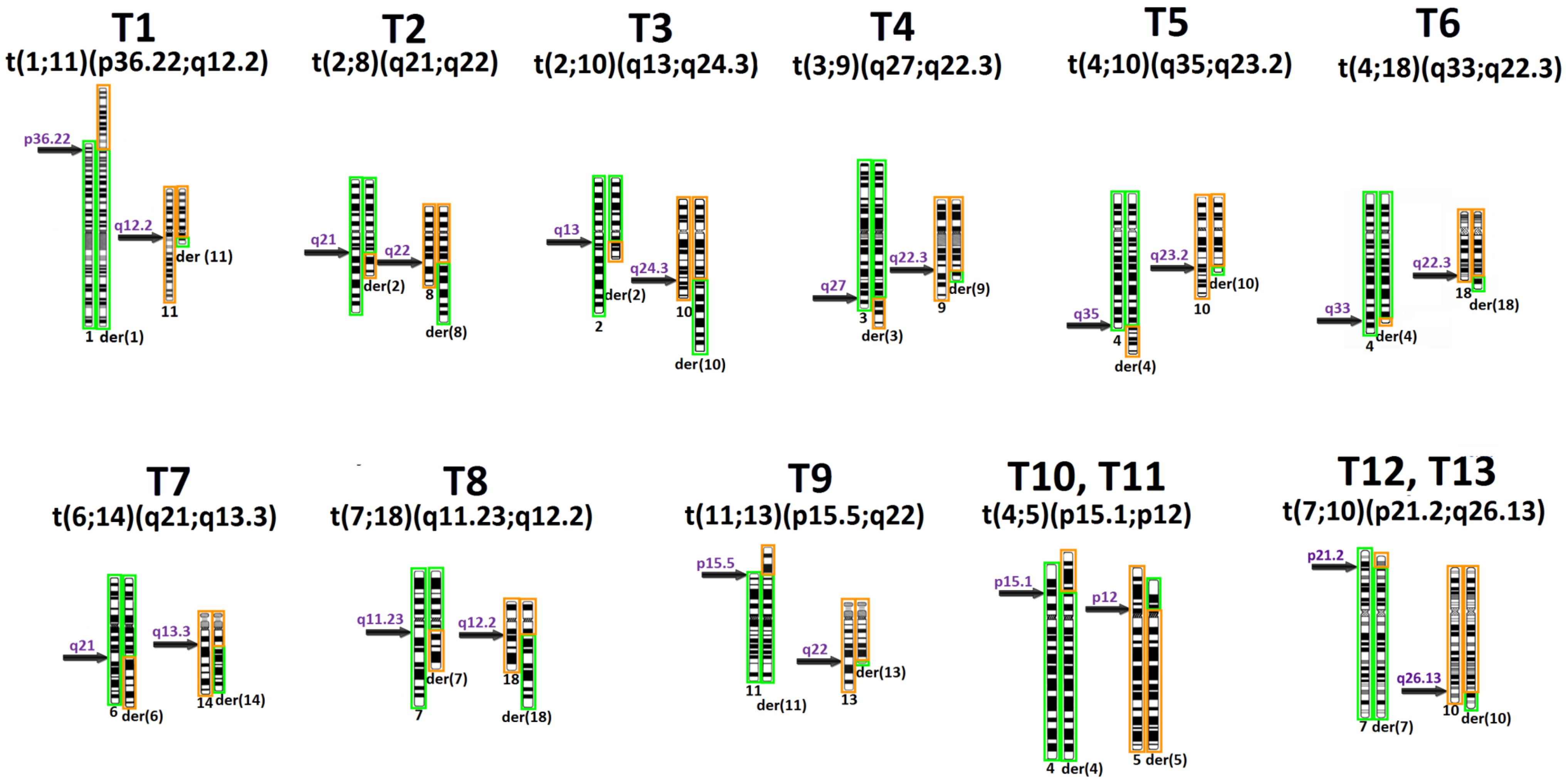

Supplement: Supplementary file 1 — Figure S1. Schematic GTG ideograms of 11 reciprocal chromosomal translocations from 13 male carriers evaluated in the study. In each RCT, breakpoints were marked with arrows, and chromosomes from each pair were differently coloured to show the size of translocated segments (TS). (PDF 369 kb) [file 12920_2018_470_MOESM1_ESM.pdf]
