# Supplementary material for: Chromosome (re)positioning in spermatozoa of fathers and sons – carriers of reciprocal chromosome translocation (RCT)
Source: BMC Med Genomics. 2019 Feb 1;12:30. doi: 10.1186/s12920-018-0470-7 (PMC6359769; doi:10.1186/s12920-018-0470-7)
Supplement: Supplementary file 2 — Table S1. Chromosome characteristics in each of analyzed RCTs. TS – translocated fragment (from breakpoint to the end of a chromosomal arm), IS – interstitial fragment (from centromere to the breakpoint); f – father, s – son; ‘+’ – presence of a proper feature, grey colour – cases with TS values below 50% in both engaged chromosomes simultaneously. All values counted according to data from NCBI Genome Data Viewer, GRCh38.p11 (https://www.ncbi.nlm.nih.gov/genome/gdv/). (PDF 578 kb) [file 12920_2018_470_MOESM2_ESM.pdf]

Additional file 2: Table S1. Chromosome characteristics in each of analyzed RCTs. TS – translocated fragment (from breakpoint to the end of a chromosomal arm), IS – interstitial fragment (from centromere to the breakpoint); f – father, s – son; '+' – presence of a proper feature, grey colour – cases with TS values below 50% in both engaged chromosomes simultaneously. All values counted according to data from NCBI Genome Data Viewer, GRCh38.p11 (https://www.ncbi.nlm.nih.gov/genome/gdv/)

no. of repositioned chromosomes	RCT			breakpoint near centromere	breakpoint near telomere	acrocentric presence	opposite arms	length of the chromosome [Mpz]	centromere position [Mpz]	breakpoint position [Mpz]	TS length [Mpz]	TS length [% of arm]	IS length [Mpz]	IS length [% of arm]	IS length [% of chr]
9/9	T7	t(6;14)	6q21			+		130.8	59.2	109.6	21.2	29.61	50.4	70.39	38.53
4,7,8,9,10,11,18,X,Y			14q13.3	+				107.0	17.2	36.5	70.5	78.51	19.3	21.49	18.04
8/9	T10	t(4;5) f	4p15.1					190.2	50.7	31.6	31.6	62.33	19.1	37.67	10.04
4,7,9,10,11,18,X,Y			5p12	+				181.5	48.3	43.5	43.5	90.06	4.8	9.94	2.64
7/9	T1	t(1;11)	1p36.22		+		+	249.0	123.5	11.0	11.0	8.91	112.5	91.09	45.18
4,7,8,9,18,X,Y			11q12.2	+				135.1	53.0	60.0	75.1	91.47	7.0	8.53	5.18
7/9	T13	t(7;10) s	7p21.2				+	159.3	60.0	9.5	9.5	15.83	50.5	84.17	31.70
4,8,10,11,18,X,Y			10q26.13					133.8	40.2	124.0	9.8	10.47	83.8	89.53	62.63
6/9	T12	t(7;10) f	7p21.2				+	159.3	60.0	9.5	9.5	15.83	50.5	84.17	31.70
4,8,10,18,X,Y			10q26.13					133.8	40.2	124.0	9.8	10.47	83.8	89.53	62.63
6/9	T11	t(4;5) s	4p15.1					190.2	50.7	31.6	31.6	62.33	19.1	37.67	10.04
4,7,9,11,X,Y			5p12	+				181.5	48.3	43.5	43.5	90.06	4.8	9.94	2.64
6/9	T2	t(2;8)	2q21					242.2	93.2	138.5	103.7	69.60	45.3	30.40	18.70
7,8,10,11,X,Y			8q22					145.1	44.6	99.0	46.1	45.87	54.4	54.13	37.49
6/9	T8	t(7;18)	7q11.23	+				159.3	60.0	75.3	84.0	84.59	15.3	15.41	9.60
4,7,8,10,11,X			18q12.2	+				80.4	18.4	37.2	43.2	69.68	18.8	30.32	23.38
5/9	T3	t(2;10)	2q13	+				242.2	93.2	110.0	132.2	88.72	16.8	11.28	6.94
4,9,10,11,Y			10q24.3					133.8	40.2	102.0	31.8	33.97	61.8	66.03	46.19
5/9	T9	t(11;13)	11p15.5		+	+	+	135.1	53.0	1.7	1.7	3.21	51.3	96.79	37.97
7,9,10,18,X			13q22					114.4	17.0	75.5	38.9	39.94	58.5	60.06	51.14
4/9	T5	t(4;10)	4q35		+			190.2	50.7	186.0	4.2	3.01	135.3	96.99	71.14
11,18,X,Y			10q23.2					133.8	40.1	87.1	46.7	49.84	47.0	50.16	35.13
4/9	T6	t(4;18)	4q33		+			190.2	50.7	170.0	20.2	14.48	119.3	85.52	62.72
4,10,X,Y			18q22.3		+			80.4	18.4	73.0	7.4	11.94	54.6	88.06	67.91
3/9	T4	t(3;9)	3q27		+			198.3	92.0	185.5	12.8	12.04	93.5	87.96	47.15
4,9,11			9q22.3					138.4	44.5	95.5	42.9	45.69	51.0	54.31	36.85
